# Supplementary material for: Multiple roles for hypoxia inducible factor 1-alpha in airway epithelial cells during mucormycosis
Source: Nat Commun. 2024 Jun 20;15:5282. doi: 10.1038/s41467-024-49637-8 (PMC11190229; doi:10.1038/s41467-024-49637-8)
Supplement: Supplementary file 1 — Supplementary Information [file 41467_2024_49637_MOESM1_ESM.pdf]

Supplementary Figure 1

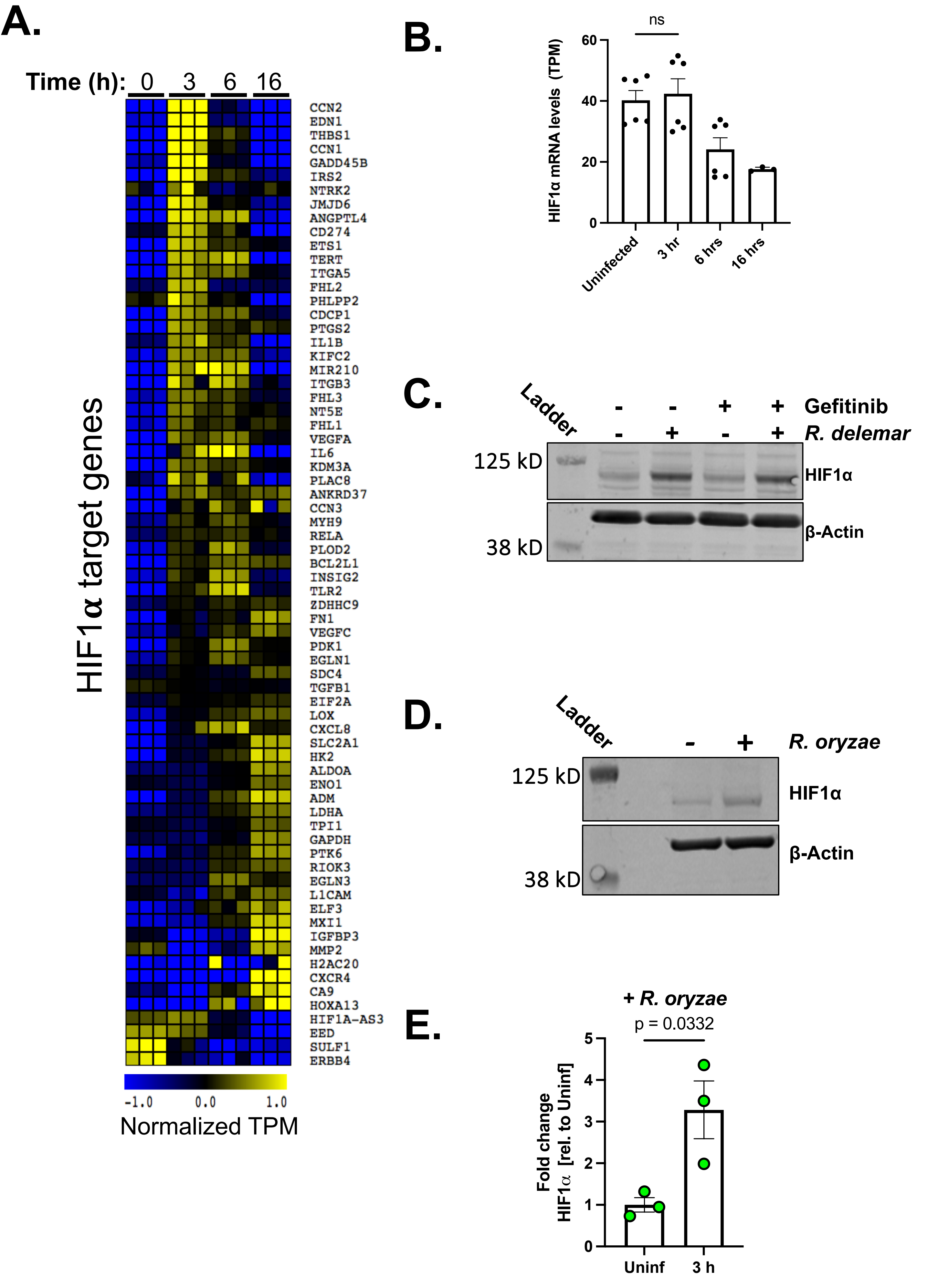

Supplementary Figure 1. Activation the HIF1α pathway by Mucorales. (A) Expression of known HIF1α-target genes. Plotted are log transformed TPM values that have been normalized across all 12 samples. Yellow indicates high gene expression; blue indicates low expression. Each column represents an individual sample. (B) Expression of HIF1**α** mRNA over the time course of HSAEC1-KT infection with *R. delemar*. Data represents the mean ± SEM of two independent experiments performed in triplicate for uninfected, 3 h, and 6 h groups (n = 6). n = 3 for the 16 h group. Two-tailed Student t-test was used for statistical analysis. ns, not significant. (C) Immunoblot representing HIF1α accumulation from whole cell lysates collected 3 hours post-infection of HSAEC1-KT cells with *R. delemar* in the presence or absence of EGFR inhibitor, Gefitinib (25 μM). (D) Immunoblot representing HIF1α accumulation from whole cell lysates collected 3 hours post-infection of HSAEC1-KT cells with *R. oryzae*. (E) Densitometric analysis of the immunoblot in panel D. Data represents the mean ± SEM of three independent experiments performed in singlicate. Two-tailed Student t-test was used for statistical analysis.

Supplementary Figure 2

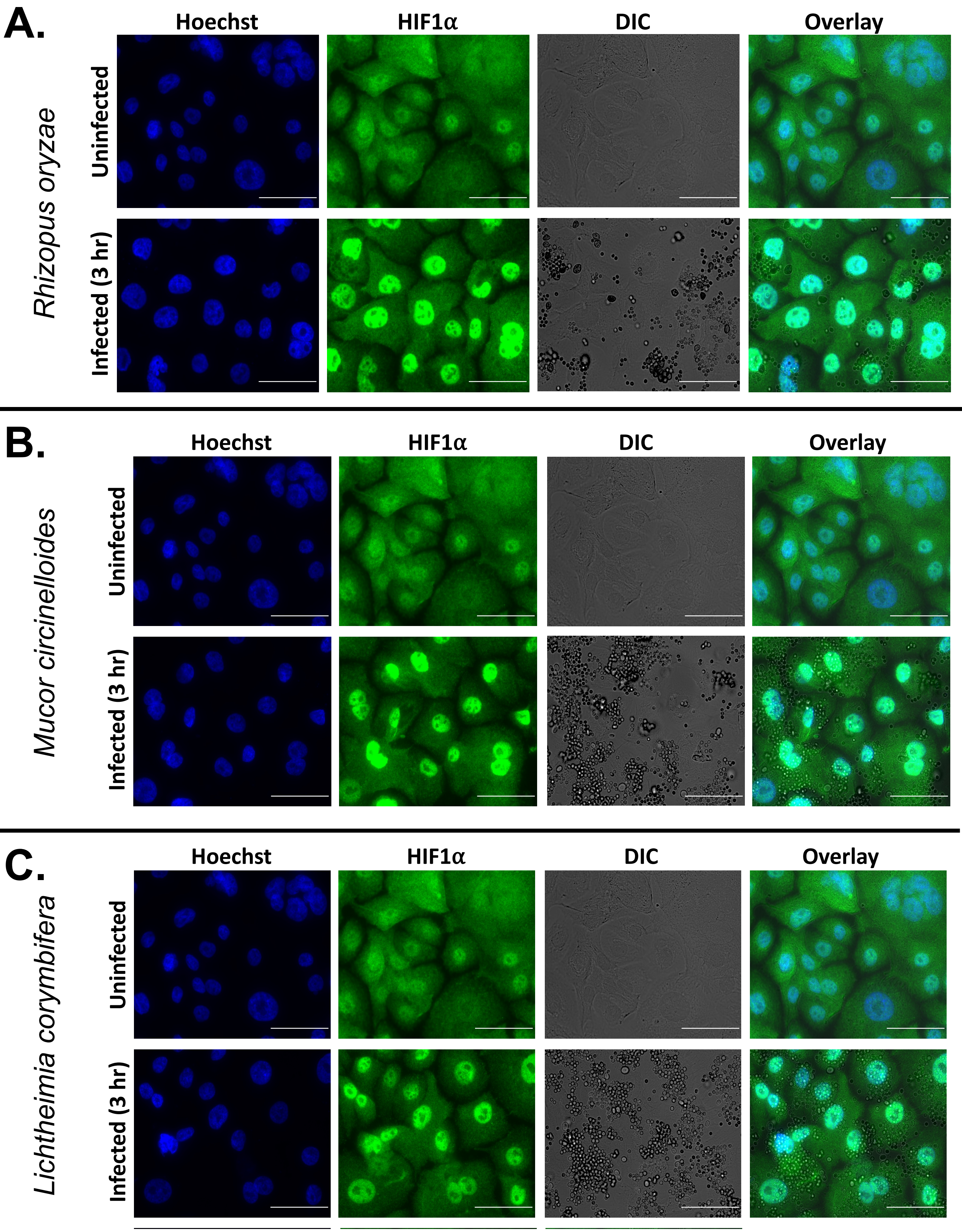

Supplementary Figure 2. HIF1α localization during *in vitro* infection with Mucorales. HSAEC1-KT cells were infected with (A) *R. oryzae*, (B) *Mucor circinelloides*, or (C) *Lichtheimia corymbifera* for 3 h and localization of HIF1α was assessed by indirect immunofluorescence with an anti-HIF1α antibody (green). Hoechst stain was used to visualize nuclei (blue). DIC, differential inference contrast. Scale bars = 100 µm. Microscopy experiments were performed two times independently with similar results.

Supplementary Figure 3

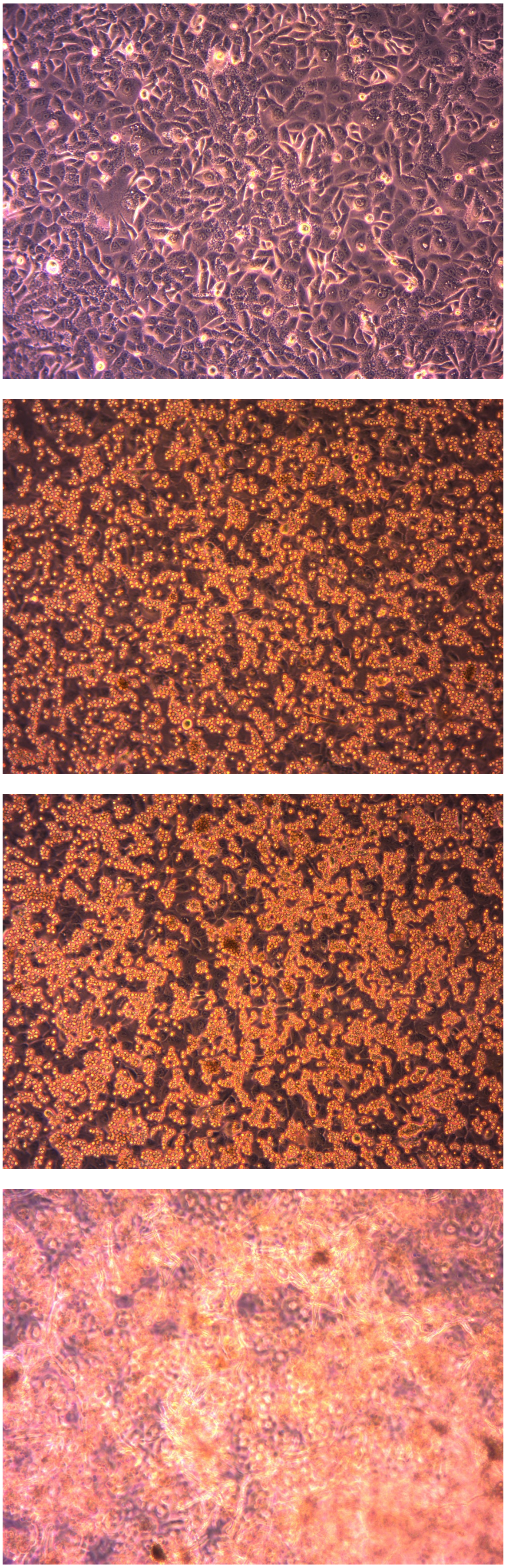

Uninfected

3 hours

6 hours

16 hours

Supplementary Figure 3. Interaction of *R. delemar* with HSAEC1-KT cells.

Microscopic images of HSAEC1-KT cells infected with each *R. delemar* for 3, 6, or 16 h *in vitro*. All pictures were taken with the 10X objective. Experiments were performed three times independently with similar results.
