# Supplementary material for: Multiple roles for hypoxia inducible factor 1-alpha in airway epithelial cells during mucormycosis
Source: Nat Commun. 2024 Jun 20;15:5282. doi: 10.1038/s41467-024-49637-8 (PMC11190229; doi:10.1038/s41467-024-49637-8)
Supplement: Supplementary file 3 — Description of Additional Supplementary Files [file 41467_2024_49637_MOESM3_ESM.pdf]

Description of Additional Supplementary Files

Supplementary Data 1. RNA-seq mapping statistics for each sample.

Supplementary Data 2. Differentially expressed host genes after 3 hours of infection.

Supplementary Data 3. Differentially expressed host genes after 6 hours of infection.

Supplementary Data 4. Differentially expressed host genes after 16 hours of infection.

Supplementary Data 5. Upstream Regulator analysis of host pathways in response to *R. delemar* infection.

Supplementary Data 6. Normalized read counts for each sample in RNA-seq experiment #1.

Supplementary Data 7. LW6-sensitive gene expression following 3 hours of infection.

Supplementary Data 8. Normalized read counts for each sample in RNA-seq experiment #2.
