# Supplementary material for: Multiple roles for hypoxia inducible factor 1-alpha in airway epithelial cells during mucormycosis
Source: Nat Commun. 2024 Jun 20;15:5282. doi: 10.1038/s41467-024-49637-8 (PMC11190229; doi:10.1038/s41467-024-49637-8)
Supplement: Supplementary file 12 — Reporting Summary [file 41467_2024_49637_MOESM12_ESM.pdf]

nature portfolio

Corresponding author(s): Vincent Bruno

Last updated by author(s): Jun 4, 2024

Reporting Summary

Nature Portfolio wishes to improve the reproducibility of the work that we publish. This form provides structure for consistency and transparency in reporting. For further information on Nature Portfolio policies, see our Editorial Policies and the Editorial Policy Checklist.

Statistics

For all statistical analyses, confirm that the following items are present in the figure legend, table legend, main text, or Methods section.

n/a	Confirmed	
☐	☒	The exact sample size (*n*) for each experimental group/condition, given as a discrete number and unit of measurement
☐	☒	A statement on whether measurements were taken from distinct samples or whether the same sample was measured repeatedly
☐	☒	The statistical test(s) used AND whether they are one- or two-sided<br>*Only common tests should be described solely by name; describe more complex techniques in the Methods section.*
☒	☐	A description of all covariates tested
☒	☐	A description of any assumptions or corrections, such as tests of normality and adjustment for multiple comparisons
☐	☒	A full description of the statistical parameters including central tendency (e.g. means) or other basic estimates (e.g. regression coefficient) AND variation (e.g. standard deviation) or associated estimates of uncertainty (e.g. confidence intervals)
☐	☒	For null hypothesis testing, the test statistic (e.g. *F*, *t*, *r*) with confidence intervals, effect sizes, degrees of freedom and *P* value noted<br>*Give P values as exact values whenever suitable.*
☒	☐	For Bayesian analysis, information on the choice of priors and Markov chain Monte Carlo settings
☒	☐	For hierarchical and complex designs, identification of the appropriate level for tests and full reporting of outcomes
☒	☐	Estimates of effect sizes (e.g. Cohen's *d*, Pearson's *r*), indicating how they were calculated

Our web collection on statistics for biologists contains articles on many of the points above.

Software and code

Policy information about availability of computer code

Data collection	No software was used.
Data analysis	HISAT2 was used to align the RNA-seq reads to the human genome. DEseq was used to calculate differential gene expression.<br>GraphPad Prism (version 10.1.0 (264) was used for statistical analyses

For manuscripts utilizing custom algorithms or software that are central to the research but not yet described in published literature, software must be made available to editors and reviewers. We strongly encourage code deposition in a community repository (e.g. GitHub). See the Nature Portfolio guidelines for submitting code & software for further information.

Data

Policy information about availability of data

All manuscripts must include a data availability statement. This statement should provide the following information, where applicable:

- Accession codes, unique identifiers, or web links for publicly available datasets
- A description of any restrictions on data availability
- For clinical datasets or third party data, please ensure that the statement adheres to our policy

Data Availability. All of the raw sequencing reads from this study are now available at the NCBI Sequence Read Archive (SRA) under BioProject accession number PRJNA986553. The specific accession numbers for both the biosamples and raw datasets are listed in Supplementary Table 1.

Research involving human participants, their data, or biological material

Policy information about studies with human participants or human data. See also policy information about sex, gender (identity/presentation), and sexual orientation and race, ethnicity and racism.

Reporting on sex and gender	This study did not involve human participants
Reporting on race, ethnicity, or other socially relevant groupings	This study did not involve human participants
Population characteristics	This study did not involve human participants
Recruitment	This study did not involve human participants
Ethics oversight	This study did not involve human participants

Note that full information on the approval of the study protocol must also be provided in the manuscript.

Field-specific reporting

Please select the one below that is the best fit for your research. If you are not sure, read the appropriate sections before making your selection.

☒ Life sciences ☐ Behavioural & social sciences ☐ Ecological, evolutionary & environmental sciences

For a reference copy of the document with all sections, see nature.com/documents/nr-reporting-summary-flat.pdf

Life sciences study design

All studies must disclose on these points even when the disclosure is negative.

Sample size	For RNA-seq and qRT-PCR, each experimental group contained 3 samples. For cytokine analyses we performed 2 independent experiments in triplicate. Immunoblotting was done as 3 independent experiments performed in singlicate. No statistical tests were utilized to pre-determine sample size. As it is the minimum number of replicates required for inferential analysis, at least three biological replicates were utilized for all experiments.
Data exclusions	No data were excluded.
Replication	All attempts at replication were successful.
Randomization	Samples were randomly allocated into experimental groups.
Blinding	Blinding was performed for the animal experiments. For other experiment, blinding was not relevant.

Reporting for specific materials, systems and methods

We require information from authors about some types of materials, experimental systems and methods used in many studies. Here, indicate whether each material, system or method listed is relevant to your study. If you are not sure if a list item applies to your research, read the appropriate section before selecting a response.

Materials & experimental systems

n/a	Involved in the study	
☐	☒	Antibodies
☐	☒	Eukaryotic cell lines
☒	☐	Palaeontology and archaeology
☐	☒	Animals and other organisms
☒	☐	Clinical data
☒	☐	Dual use research of concern
☒	☐	Plants

Methods

n/a	Involved in the study	
☒	☐	ChIP-seq
☒	☐	Flow cytometry
☒	☐	MRI-based neuroimaging

Antibodies

Antibodies used	rabbit anti-HIF1a (Proteintech, Catalog #:20960-1-AP), 1:1000 dilution

Antibodies used
AlexaFluor 488 labeled goat anti-rabbit IgG antibody (ThermoFisher)
Biotin-labeled mouse anti-HIF1▯ monoclonal antibody (Bio-Techne Corporation)
anti-▯-actin (8H10D10), the anti-ERK1/2 (137F5), and anti-phospho-ERK1/2 (D13.14.4E) antibodies were purched from Cell Signaling Technologies.

Validation
None. All antibodies were purchased from reputable, well-established life science companies that perform extensive testing of antibodies on multiple cell lines with multiple applications.

Eukaryotic cell lines

Policy information about cell lines and Sex and Gender in Research

Cell line source(s)
HSAEC1-KT cells (CRL-4050, ATCC), male
A549 (CCL-185, ATCC), male

Authentication
None of the cell lines were authenticated

Mycoplasma contamination
Cell lines tested negative for mycoplasma contamination

Commonly misidentified lines
(See ICLAC register)
not relevant

Animals and other research organisms

Policy information about studies involving animals; ARRIVE guidelines recommended for reporting animal research, and Sex and Gender in Research

Laboratory animals
Male ICR mice (≥20 g), All mice were housed in our animal facility according to IACUC guidelines with ambient room temperature (20–26 °C), humidity (50-70%) and dark / light cycle (12 hours).

Wild animals
none

Reporting on sex
Only male mice were used

Field-collected samples
none

Ethics oversight
All animal studies were approved by the Institutional Animal Care and Use Committee (IACUC) of the Los Angeles Biomedical Research Institute at Harbor-UCLA Medical Center according to the NIH guidelines for animal housing and care (approval reference no. 21125).

Note that full information on the approval of the study protocol must also be provided in the manuscript.

Plants

Seed stocks
not relevant

Novel plant genotypes
not relevant

Authentication
not relevant
